# Digital Intervention to Improve Health Services for Young People in Zimbabwe: Process Evaluation of ‘Zvatinoda!’ (What We Want) Using the RE-AIM (Reach, Effectiveness, Adoption, Implementation, and Maintenance) Framework

**DOI:** 10.2196/53034

**Published:** 2024-09-24

**Authors:** Constance Ruth Sina Mackworth-Young, Privillage Charashika, Zvatinoda Youth Advisory Group, Leyla Larsson, Olivia Jane Wilding-Davies, Nikita Simpson, Anna Sorrel Kydd, Theonevus Tinashe Chinyanga, Rashida Abbas Ferrand, Aveneni Mangombe, Karen Webb, Aoife Margaret Doyle

**Affiliations:** 1 Department of Global Health and Development London School of Hygiene and Tropical Medicine London United Kingdom; 2 The Health Research Unit Zimbabwe Biomedical Research and Training Institute Harare Zimbabwe; 3 Organization for Public Health Interventions and Development Harare Zimbabwe; 4 See Acknowledgments; 5 Department of Anthropology and Sociology School of Oriental and African Studies University of London London United Kingdom; 6 The SHM Foundation London United Kingdom; 7 Department of Clinical Research, London School of Hygiene & Tropical Medicine London United Kingdom; 8 Ministry of Health and Child Care Harare Zimbabwe; 9 International Statistics and Epidemiology Group London School of Hygiene & Tropical Medicine London United Kingdom

**Keywords:** adolescents, young people, digital health, mobile intervention, HIV, sexual and reproductive health, Zimbabwe

## Abstract

**Background:**

Youth in Southern Africa face a high burden of HIV and sexually transmitted infections, yet they exhibit low uptake of health care services.

**Objective:**

The Zvatinoda! intervention, co-designed with youth, aims to increase the demand for and utilization of health services among 18-24-year-olds in Chitungwiza, Zimbabwe.

**Methods:**

The intervention utilized mobile phone–based discussion groups, complemented by “ask the expert” sessions. Peer facilitators, supported by an “Auntie,” led youth in anonymous online chats on health topics prioritized by the participants. Feedback on youth needs was compiled and shared with health care providers. The intervention was tested in a 12-week feasibility study involving 4 groups of 7 youth each, totaling 28 participants (n=14, 50%, female participants), to evaluate feasibility and acceptability. Mixed methods process evaluation data included pre- and postintervention questionnaires (n=28), in-depth interviews with participants (n=15) and peer facilitators (n=4), content from discussion group chats and expert guest sessions (n=24), facilitators’ debrief meetings (n=12), and a log of technical challenges. Descriptive quantitative analysis and thematic qualitative analysis were conducted. The RE-AIM (Reach, Effectiveness, Adoption, Implementation, and Maintenance) framework was adapted to analyze and present findings on (1) reach, (2) potential efficacy, (3) adoption, (4) implementation, and (5) maintenance.

**Results:**

Mobile delivery facilitated engagement with diverse groups, even during COVID-19 lockdowns (reach). Health knowledge scores improved from pre- to postintervention across 9 measures. Preintervention scores varied from 14% (4/28) for contraception to 86% (24/28) for HIV knowledge. After the intervention, all knowledge scores reached 100% (28/28). Improvements were observed across 10 sexual and reproductive health (SRH) self-efficacy measures. The most notable changes were in the ability to start a conversation about SRH with older adults in the family, which increased from 50% (14/28) preintervention to 86% (24/28) postintervention. Similarly, the ability to use SRH services even if a partner does not agree rose from 57% (16/28) preintervention to 89% (25/28) postintervention. Self-reported attendance at a health center in the past 3 months improved from 32% (9/28) preintervention to 86% (24/28) postintervention (potential efficacy). Chat participation varied, largely due to network challenges and school/work commitments. The key factors facilitating peer learning were interaction with other youth, the support of an older, knowledgeable “Auntie,” and the anonymity of the platform. As a result of COVID-19 restrictions, regular feedback to providers was not feasible. Instead, youth conveyed their needs to stakeholders through summaries of key themes from chat groups and a music video presented at a final in-person workshop (adoption and implementation). Participation in discussions decreased over time. To maintain engagement, introducing an in-person element was suggested (maintenance).

**Conclusions:**

The Zvatinoda! intervention proved both acceptable and feasible, showing promise for enhancing young people’s knowledge and health-seeking behavior. Potential improvements include introducing in-person discussions once the virtual group has established rapport and enhancing feedback and dialog with service providers.

## Introduction

Improving young people’s engagement with health services is important not only to reduce morbidity and mortality but also to enhance future health and well-being [1,2]. The relatively low demand for health services, combined with poor acceptability and accessibility, results in a lower uptake of preventive and treatment services among young people compared with children and adults [2,3]. In Zimbabwe, 31.9% of the population is aged 10-25 years [4]. Young people’s health needs include preventive services (eg, young women are most at risk for new HIV infections), treatment services, and reproductive services (22% of 15-19-year-olds have started childbearing) [5]. However, barriers to health service accessibility for young people in Zimbabwe are user fees (currently approximately US $5 per consultation at urban municipal clinics), overburdened clinics, health care providers perceived as judgmental, a tendency to seek health care only when ill, and fear of diagnosis [6,7].

Despite the recognition of the importance of youth-friendly health services 2 decades ago [8], progress has been slow [9,10]. Communication between young people and health care providers regarding their health needs remains minimal [11]. In Zimbabwe, Health Centre Committees, comprising key community leaders and stakeholders (including local and traditional leaders, village health workers, teachers, and older individuals), strengthen community-facility linkages and provide a platform to raise community preferences and needs for high-quality health care services [12]. Despite being an underserved and growing stakeholder group, young people are underrepresented in such forums, and no formal mechanism exists to provide feedback on their needs and preferences to health care providers and decision makers.

Digital health interventions, including online or phone support, are an increasingly common method to connect young people to sexual and reproductive health (SRH) information and services [13]. Systematic reviews of young people’s health have concluded that targeted digital media interventions have the potential to improve SRH knowledge and access to SRH services. However, a greater understanding of the implementation and cost-effectiveness of these interventions is needed [14,15]. The potential for digital health interventions in Zimbabwe has increased since 2020, as COVID-19 restrictions constrained in-person interventions and interactions, while young people’s health needs did not decrease. In 2018, phone ownership among 13-24-year-olds in Harare was 63%, with 11% of those without a phone reporting using a shared phone [16]. Phone ownership increased with age, reaching 72% among 18-19-year-olds and 85% among 20-24-year-olds [16]. Recommendations for the development of digital health interventions underscore the importance of young people’s input to ensure the success of health interventions targeting them, including involving young people from the design phase [17]. Co-designing interventions with young people increases the likelihood of acceptability and uptake [18].

The Zvatinoda! (“What we want!” in Shona) study aimed to co-design an intervention with young people (aged 18-24 years) and key adults in their lives to improve both the demand for and uptake of essential health services by young people in Zimbabwe. This paper reports on the process evaluation of the Zvatinoda! feasibility study. The process evaluation assessed the intervention’s reach, potential efficacy, adoption, implementation, and maintenance, drawing from the RE-AIM (Reach, Effectiveness, Adoption, Implementation, and Maintenance) framework [19,20].

## Methods

### Study Design

The feasibility study of the Zvatinoda! intervention was conducted over 12 weeks, from July to October 2021.

### Participants

A total of 28 participants were recruited from the geographical catchment area of Zengeza and Seke North municipal clinics, located in Chitungwiza, a high-density suburb of Harare, Zimbabwe. Zengeza and Seke North are periurban satellite communities characterized by fragmented and mixed urban and rural neighborhoods, with both formal and informal housing settlements. Participants were initially recruited at youth hangouts and then through snowball sampling to purposefully include a diverse group of young people (aged 18-24 years), considering factors such as age, gender, HIV status, and disability.

### Zvatinoda! Intervention

The intervention consisted of mobile phone–based discussion groups tested with 4 groups of 7 young people each, using Rocket.Chat (Rocket.Chat Holdings, Ltd.), a messaging platform that allows users to choose usernames (pseudonyms) for anonymity [21]. The intervention aimed to both generate service demand among young people and facilitate communication between them and health system stakeholders to improve the quality of health services for young people.

### Zvatinoda! Intervention Co-Design

The intervention was adapted from 2 previously trialed models that used anonymous mobile chats to provide peer-to-peer psychosocial support or involved an ‘Auntie’ who offered confidential information, counseling, and health service referrals in Zimbabwe [22-25]. The adapted intervention was co-designed with peer facilitators as well as key community and health system stakeholders. The co-design process included (1) community mapping through transect walks to identify risk “hotspots” and available health services; (2) in-depth interviews with young people and health service providers; (3) participatory action research workshops with 25 young people at each of the 2 study sites to prioritize health topics for the discussion curriculum, determine implementation modalities of mobile chat groups (including frequency, facilitation, and safeguarding), and identify preferred methods for providing feedback to health system stakeholders; and (4) a participatory action research workshop with 6 health care workers.

The 8 peer facilitators were selected through a 4-stage process involving (1) community sensitization about the Zvatinoda! project and the role of the peer facilitators; (2) self-nomination by young people aged 18-24 years as candidates; (3) short oral presentations by candidates describing their skills, strengths, and interest in peer facilitation; and (4) community voting to select the 8 peer facilitators, conducted by community and health system stakeholders (n=7) and young people (n=39) [26].

### Intervention Delivery

Each mobile phone–based discussion group was facilitated by 2 peer facilitators (aged 19-24) and supervised by 1 adult research team member to address any harmful discussion content. Participants collectively decided on the best time for the mobile chat group discussions.

The discussion curriculum, developed during the participatory action research workshops, focused each week on a different health-related topic. Topics included relationships, pregnancy, family planning, sexually transmitted infections, HIV, cervical cancer, male circumcision, sexual abuse, substance abuse, mental health, gender-based violence, personal hygiene, and COVID-19. An adult guest expert joined each week for a live 1-hour chat session, during which participants could ask questions anonymously. Individuals who were unable to attend the expert chat sessions or who experienced network issues could access the discussion content later.

To include youth participants who did not have phones, all participants were provided with project phones, which could only be used for the Rocket.Chat app, and a data allowance for the duration of the intervention.

Monthly in-person and virtual feedback sessions with health care workers and community members were planned. During the co-design process, young people expressed a preference for direct feedback mechanisms to health care professionals rather than mediated feedback. A final face-to-face participatory workshop with peer facilitators, health care providers, and community members was planned to discuss young people’s health needs and potential improvements to health service delivery.

### Mixed Methods Process Evaluation Data Collection

The feasibility study was evaluated using a mixed methods process evaluation. The following data sources were utilized: quantitative pre- and postintervention questionnaires (n=28), in-depth interviews with participants (n=15) and peer facilitators (n=4), discussion group chats and expert guest sessions (n=24), facilitator debrief meetings (n=12), and a log of technical challenges.

A pre- and postintervention questionnaire, programmed in Open Data Kit (Get ODK), was self-completed by all 28 participants on tablets at the beginning and end of the 12-week feasibility study. A research staff member was present to assist with any queries. The questionnaire assessed participants’ experiences with the discussion groups and measured key outcomes, including SRH literacy, SRH self-efficacy, SRH service uptake, and perceptions of the youth-friendliness of services. Additional questions on sources of SRH information, aspirations, self-esteem, and perceived social support were included in the questionnaire but are not reported in this paper.

We also invited 15 participants to participate in in-depth interviews to understand their experiences with the Zvatinoda! intervention. These interviews aimed to explore their engagement with the program, perceptions of the curriculum topics, facilitators, expert sessions, their experiences with the phones, and the perceived impact of the intervention on both themselves and young people’s health services. Interview participants were purposively selected to represent a range of ages, genders, participation levels from across the 4 groups (including both active and quieter participants), and any individuals with particularly interesting characteristics or stories. Additionally, 4 peer facilitators participated in in-depth interviews with aims similar to those of the participant interviews, but with a focus on their experiences of facilitation. These interviews were conducted by a trained qualitative researcher; lasted between 30 and 60 minutes; and were audio recorded, transcribed, and translated.

The discussion group chats were downloaded and analyzed weekly in analysis reports. These reports aimed to identify key themes discussed, record the level and type of interaction within the group, assess whether discussions remained on the agreed weekly topic, and note any challenges encountered. Each week, 2 out of the 4 groups were analyzed in detail on an alternating basis, and a higher-level analysis report summarizing the main themes from all 4 groups was prepared. The process of delivering the intervention was documented through notes from weekly facilitator debrief meetings held during the feasibility study. Additionally, a log of technical challenges was maintained throughout the study.

### Data Analysis

We adapted the RE-AIM framework for analysis to assess the (1) reach, (2) potential efficacy, (3) adoption, (4) implementation, and (5) maintenance of the intervention within the feasibility study [19,20]. For reach, we evaluated the diversity of participants and the potential reach of the intervention if scaled. Given the small scale of the feasibility study, we focused on potential efficacy rather than effectiveness to assess the intervention’s impact on key outcomes for the 28 participants. For adoption, we adapted the RE-AIM framework’s focus from settings that adopt the intervention to assess adoption among participants, which was more relevant to this feasibility study. For implementation, we modified the framework to evaluate factors affecting intervention acceptability, fidelity to the intervention protocol, and the delivery of the intervention. For maintenance, we assessed the potential for the intervention to have a long-term impact.

Qualitative data were coded using the 5 adapted pillars of the RE-AIM framework as main deductive themes. After familiarizing ourselves with a subset of the qualitative data, inductive subthemes were developed. For each main theme, the subthemes were introduced (Textbox 1). The entire qualitative data set was coded by CRSMY using the deductive main themes and inductive subthemes, with codes remaining flexible throughout the coding process in NVivo (Lumivero; formerly QSR International). An analysis report was prepared by CRSMY and reviewed with PC, KW, and AD to assess consensus and refine the findings. This report formed the basis for the results presented in this paper. Additional quotes for each theme and subtheme are provided.

**Subthemes.**

- Reach

The reach of mobile interventions and representativeness.

- Potential Efficacy

Sexual and reproductive health knowledge acquired, changes in behavior, and spreading knowledge to others.

- Adoption

Challenges with mobile networks and variability in participation

- Implementation

Participants’ enjoyment of the Zvatinoda! groups, aspects of the intervention that participants valued (eg, group interaction, privacy, having project phones, and discussions being young people led and adult supported), challenges in delivering regular feedback to health care providers, and peer facilitators’ personal development through the intervention

- Maintenance

Reduced engagement at the end of the intervention and a desire for an in-person element.

Participant sociodemographic characteristics were described. Quantitative data from the pre- and postintervention questionnaires were analyzed and reported as the number and proportion of participants providing desired responses to questions on the following topics: SRH knowledge (5 questions), knowledge of SRH services (4 questions), self-efficacy in communicating about SRH (3 questions), access to SRH services and information (5 questions), condom use (2 questions), health-seeking behavior in the past 3 months (1 question), and youth-friendliness of health services during their last visit in the past 3 months (7 questions). Overall satisfaction with the health facility was measured on a scale from 0 (worst) to 10 (best) and presented as a mean score [27]. Knowledge questions were adapted from the Insaka HIV Knowledge Quiz [22] and the Adolescents 360 Evaluation Questionnaire [28], while youth-friendliness [27] and stigma questions [29] were also adapted. Quantitative analysis was conducted using Stata (version 16.1; StataCorp).

### Ethical Considerations

Ethics approval was obtained from the Medical Research Council of Zimbabwe (MRCZ/A/2563) and the London School of Hygiene and Tropical Medicine (LSHTM 18057). Permissions were also obtained from the Ministry of Health and Child Care, the Chitungwiza City Health Ethical Review Board, and the District Administrator’s Office. Written informed consent was obtained from all research participants, and all data were anonymized before analysis. Participants received a travel reimbursement of US $2-5 for attending data collection sessions.

## Results

### Reach

The intervention pilot involved 7 participants in each of 4 groups, totaling 28 participants. During the COVID-19 pandemic, the mobile-based nature of the intervention allowed it to reach young people when physical gatherings were restricted. Participants were purposively selected based on a range of characteristics, including age (18-24 years), gender, HIV status, parenting status, and disability (Table 1). This diverse group was appreciated by participants for the variety of identities represented and the range of views expressed: “the fact that you managed to engage other young people like disabled young people...that alone moved me and that was a good experience” [Male peer facilitator, 24 years]. Including young people living with and not living with HIV in mixed groups was appreciated.

**Table 1:** Sociodemographic characteristics of the participants (N=28) in the Zvatinoda! intervention.

Sociodemographic characteristics			Values, n (%)	
**Sex**				
	Female	14 (50)		
	Male	14 (50)		
**Age (years)**				
	18-19	8 (29)		
	20-21	11 (39)		
	22-24	9 (32)		
**Marital status**				
	Single	28 (100)		
**Ever attended school**				
	Yes	28 (100)		
**Occupational status**				
	In school	14 (50)		
	Out of school—working	5 (18)		
	Out of school—not working	9 (32)		
**Highest educational level achieved**				
	Secondary	23 (82)		
	Tertiary	5 (18)		
**Religion**				
	Roman Catholic	5 (18)		
	Protestant	3 (11)		
	Pentecostal	8 (29)		
	Apostolic	2 (7)		
	Other Christian	10 (36)		
**Spoken language outside of the home**				
	Shona	24 (86)		
	English	4 (14)		

### Potential Efficacy

#### Participants Acquired SRH Knowledge

Health knowledge scores improved from pre- to postintervention across 9 measures. Preintervention scores ranged from 4 out of 28 (14%) for contraception to 24 out of 28 (86%) for HIV knowledge. After the intervention, all knowledge scores reached 100% (28/28; Table 2).

**Table 2:** Changes in SRH^a^ knowledge, knowledge of SRH services, SRH self-efficacy, and health-seeking behaviors in the pre- and postintervention questionnaire.

Questions			Prequestionnaire (N=28), n (%)		Postquestionnaire (N=28), n (%)
**SRH knowledge quiz^b^**					
	Antiretroviral drugs are medicines that help people with HIV positivity to live healthier lives	6 (21)		28 (100)	
	Using modern contraceptives to prevent unintended pregnancies is important	24 (86)		28 (100)	
	There is a way for someone who has HIV to get pregnant without passing the virus on to her child	20 (71)		28 (100)	
	A person can get HIV by sharing a glass of water with someone who has HIV	26 (93)		28 (100)	
	Changes to normal menstrual bleeding patterns, which are caused by some contraceptives, can make a girl permanently infertile	4 (14)		28 (100)	
**Knowledge of accessible SRH services^c^**					
	Do you know of a place where you can get condoms comfortably?	19 (68)		28 (100)	
	Do you know of a place where you can get access to family planning comfortably?	20 (71)		28 (100)	
	Do you know of a place where you can get an HIV test comfortably?	23 (82)		28 (100)	
	Is the local health facility a place where someone like you could go to get information?	23 (82)		28 (100)	
**Self-efficacy to communicate about SRH^d^**					
	I feel able to start a conversation with my husband/wife/boyfriend/girlfriend about SRH	26 (93)		28 (100)	
	I feel able to start a conversation with my friends about SRH	26 (93)		27 (96)	
	I feel able to start a conversation with older adults in my family about SRH	14 (50)		24 (86)	
**Self-efficacy to access SRH services and information^d^**					
	I feel able to obtain information on SRH services and products if I need to	25 (89)		28 (100)	
	I feel able to get to a place where SRH services are offered if I decide that I need them	27 (96)		28 (100)	
	I feel able to obtain SRH services and products if I need them	25 (89)		28 (100)	
	I feel able to use SRH services even if my husband/wife/boyfriend/girlfriend does not want me to	16 (57)		25 (89)	
	I feel able to use SRH services even if older adults in my family (eg, parents, guardian, aunt/uncle) do not want me to	19 (68)		25 (89)	
	I can ask a new partner to use condoms	18 (64)		28 (100)	
	I can refuse sex when I do not have a condom available	20 (71)		28 (100)	
**Health-seeking behavior^e^**					
	Attended a local health facility for own care in the last 3 months?	9 (32)		24 (86)	
**Youth-friendliness of health services (only if attended in last 3 months)^f^**					
	The last time I attended for my own care, I felt I was treated with courtesy and respect^g^	8/9 (89)		22/24 (92)	
	The last time I attended for my own care, the health care provider listened carefully^g^	7/9 (78)		22/24 (92)	
	The last time I attended for my own care, the health care provider explained things in an understandable way^g^	9/9 (100)		21/24 (88)	
	The last time I attended for my own care, I had enough time to discuss my medical problem with the health care provider^g^	6/9 (67)		19/24 (79)	
	I have been denied SRH services because of my age, marital status, or both	3/9 (33)		3/24 (13)	
	Health care workers talked badly about me because of my request for SRH services	3/9 (33)		2/24 (8)	
	A health care worker disclosed my SRH status/use of SRH services without permission	1/9 (11)		3/24 (13)	
	Satisfaction with health facility (mean rating out of 10)^h^	5.9		7.0	

^a^SRH: sexual and reproductive health.

^b^Values correspond to the number and proportion of those with correct answers.

^c^Values correspond to the number and proportion of those with knowledge of accessible services.

^d^Values correspond to the number and proportion of those who agree.

^e^Values correspond to the number and proportion of those saying ‘yes’.

^f^Values correspond to the number and proportion agreeing/strongly agreeing.

^g^Includes those agreeing/strongly agreeing.

^h^On a scale of 0-10, where 0=worst and 10=best.

Participants reported that their main likes included receiving information and learning about health services (Table 3). The value and enjoyment of the intervention largely came because participants “liked the fact that we are learning something through the groups” [Female participant, 23 years]. Many participants joined the intervention specifically “to learn, I wanted to learn, I’m hungry to learn” [Female participant, 18 years]. As one participant said, “I enjoyed because I now know that I can go to the clinic and get tested for HIV for free or get screened for cervical cancer” [Female participant, 22 years]. Discussion on commonly held beliefs about SRH was particularly helpful:

in the community there is a myth that if you use Jadelle [family planning method] before bearing children you won’t have children at all; they corrected that myth

Male participant, 21 years

Participants generally felt that the information that they learned was relevant to their personal experiences: “the topics that we discuss, these are the things that we meet in our day to day lives” [Female participant, 18 years]

**Table 3:** Experience of Zvatinoda! groups (based on postintervention questionnaire responses from participants; N=28).

Questions on experience			Male (n=14), n (%)	Female (n=14), n (%)
**What has your experience been of the Zvatinoda! groups?**				
	Very positive	12 (86)		12 (86)
	Mostly positive	2 (14)		2 (14)
	Neutral/negative	0 (0)		0 (0)
**What was the main thing about participating in the Zvatinoda! groups that you really liked?^a^**				
	Felt supported by a facilitator	2 (14)		2 (14)
	Felt supported by peers	1 (7)		0 (0)
	Got information	2 (14)		5 (36)
	Guest speakers	3 (21)		0 (0)
	Had peers to talk to	1 (7)		0 (0)
	Learned about health services	5 (36)		7 (50)
**Was there anything about participating in the Zvatinoda! groups that you did not like?**				
	Yes (messages/discussion not relevant)	1 (7)		0 (0)
	No	13 (93)		14 (100)
**What changes did you make to your life since joining the Zvatinoda! discussion groups?^a^**				
	Feel able to access services/information when I need them	6 (43)		11 (79)
	Attend health facility when I needed care	5 (36)		9 (64)
	Safer sex	7 (50)		8 (57)
	More supported in decision-making	8 (57)		5 (36)
	Better communication with a sexual partner about SRH^b^	6 (43)		6 (43)
	Took up family planning	1 (7)		5 (36)
	Better communication with family about SRH	3 (21)		3 (21)
	Have more friends	4 (29)		0 (0)
	Better diet	1 (7)		0 (0)
**Would you recommend joining a Zvatinoda! group to a friend?**				
	Definitely yes	12 (86)		13 (93)
	Probably yes	2 (14)		0 (0)
	Probably no	0 (0)		1 (7)
	Definitely no	0 (0)		0 (0)

^a^Participants could choose more than 1 option.

^b^SRH: sexual and reproductive health.

#### Participants Demonstrated Changes in Behavior Following the Intervention

Many noted positive changes in health-seeking behaviors, including an increase in the proportion of those reporting the use of a health facility for their own care in the past 3 months (9/28, 32%, preintervention vs 24/28, 86% postintervention; Table 2). Improvements were observed across 10 SRH self-efficacy measures, with the most significant changes noted in 2 areas: the ability to start a conversation about SRH with older adults in the family (preintervention: 14/28, 50% vs postintervention: 24/28, 86%) and the ability to use SRH services even if a partner does not agree (preintervention: 16/28, 57% vs postintervention: 25/28, 89%; Table 2).

Specifically, many participants reported getting an HIV test as a result of the intervention:

This topic on HIV testing it helped me because I was afraid to go and get tested. When I heard others saying that they had to go and get tested I then decided to go and get tested.

Female participant, 18 years

By sharing and hearing others’ experiences, participants were encouraged to engage in positive health-seeking behaviors:

Before the groups I was afraid to go to cancer screening, now that I have information, why it’s good for me and what can happen. I am confident to go for screening now I can make informed decisions.

Male participant, 21 years

Some participants developed increased empathy for the experiences of friends and family members affected by the issues discussed. Both participants and peer facilitators noted the long-term impact of this change: “my involvement will impact me for the whole of my life, actually” [Female peer facilitator, 23 years].

#### Participants Shared the Knowledge Gained Through Zvatinoda! With Their Peers

About 96% (27/28) reported they would definitely or probably recommend Zvatinoda! to a friend (Table 2). Additionally, participants conveyed their learning from the intervention to friends and family:

My friend once said her husband was abusing her, so when we did the topic about gender-based violence so I got some points which I shared with her, now she is in a good place she reported to the police.

Male participant, 20 years

Those who had not yet shared their learning expressed intentions and plans to do so. The process of discussing the information gained through the intervention with friends and family extended its impact beyond the participants themselves:

When we get information myself, I will go with the information I will then sit with others and discuss as youths. Then one youth will assist the other person, then that person we would have assisted will meet others and assist. So more young people will be assisted with health information from Zvatinoda!.

Male participant, 21 years

Participants reported increased self-efficacy in initiating conversations about SRH with older adults in their families, with rates rising from 50% (14/28) before the intervention to 86% (24/28) after the intervention (Table 2).

### Adoption

#### Phone Network Issues Proved Challenging

The major barrier to intervention adoption was the phone network when using the project phones and the Rocket.Chat platform, which participants described as a “big challenge” [Female participant, 18 years]. This problem led to participants feeling unable to engage fully, with both participants and sometimes peer facilitators frequently arriving late to the chats. As a result, some participants were unable to engage substantively in the intervention: “the last 2 weeks I was totally absent because of the network but I managed to catch up” [Female participant, 20 years]. Phone battery problems, power cuts, and the inability to charge phones further compounded the issue. These factors added to the challenges participants faced with the intervention (Table 4).

I think because the [Rocket.Chat] App drains power, so the battery would quickly run out of power yet there were intermittent power outages [preventing charging]

Male participant, 20 years

Some participants were able to contribute to the conversations after the planned time, but the guest sessions, which were scheduled for specific times, presented a greater challenge:

mostly I would attend meetings but guest sessions honestly, I never attended because of the network, it was very poor

Female participant, 20 years

**Table 4:** Illustrative quotes from interviews with participants and facilitators, organized by the RE-AIM^a^ framework pillars.

Framework pillars			Illustrative quote
**1. Reach**			
	Reach of digital intervention	When we are using the mobile phone, it helps us not to gather around, especially this time of Covid-19, we can infect each other when we gather around. So, when we are using the phones...online it’s safe. [Female participant, 22 years] The advantage is that we avoid meetings physically thereby avoiding getting infected by Covid 19, so phones help us avoid getting sick from COVID-19. [Female participant, 22 years]	
**2. Potential efficacy**			
	Participants acquired SRH^b^ knowledge	From the first day I joined, I can say I have been learning. That is one thing that I was promised when I was being recruited and I have been getting it, its learning from other people’s different views and I have been getting it every time that we have a meeting, by the time we finish I would have learnt something new. [Female participant, 18 years] When we were learning about cervical cancer, I was happy because I now know that if I have signs and symptoms or if I suspect that I have cervical cancer I can go and get tested...I learnt a lot, I learnt that HIV testing is done for free. I Also learnt that if you are raped you can go and report and how to go about the process...I also learnt that if I get pregnant, or if I indulge in sex and if I’m not ready to get pregnant, before 72 hours I can go and buy pills. [Female participant, 22 years] What I enjoy is the guest speakers who come at the end of the week Friday because they help us on issues that we are not clear, that we don’t know, and they clear some of the misconceptions that we tell each other in the communities. [Female participant, 23 years] Myths around HIV transmission were clarified. [Female participant, 22 years]	
	Participants changed their behavior after the intervention	Yeah, it’s more to the positive side because some of the topics like HIV testing, as for me, I was afraid to get tested but I was motivated by our guest speakers who come. [Female participant, 18 years] I learnt a lot, especially, on HIV testing, it’s very important for me to go for HIV testing. Before Zvatinoda!, I had never gone through an HIV test but during the sessions and discussion, it also pushed me to go to do the test. [Male facilitator, 23 years]	
	Participants shared knowledge gained through Zvatinoda! with peers	The topics about family planning, some of my friends have been experiencing that. I have lost many friends due to pregnancies; I think the topic about family planning the guest speaker helped me so much now I know I can share information to other peers...the youths are facing a lot in the ghetto, there is drug abuse, there is gender-based violence there is sexual assault. So if you go out there and share with them and talk to them it will help them one day...About HIV testing I talked to my other boys from rugby and family, the girls from my church, what we discussed in our chat group. That program is good it taught us a lot, I can see myself helping others, not just in the Zvatinoda! project, but in the community at large. [Male participant, 20 years] I shared this one of cervical cancer to my friends. I told my friends whilst we are in class during break time. I said “guys do know that cervical cancer is something that is this” and they said, “No what is it”. I explained everything that I learnt from the discussions. [Female participant, 22 years] The youth asked me about sexually transmitted infections and engagement in sexual intercourse. So, I shared all the necessary information with them. I also share the information with the upcoming junior councillors. So, I shared with them like the issue of sexually transmitted infections and teen pregnancies. I inform them from the experience and knowledge that I have acquired from the RocketChat. [Male participant, 20 years]	
**3. Adoption**			
	Phone network proved challenging	The only challenge I can say is the network challenge, most of us have been facing this challenge. [Female participant, 18 years] Ok I do not want to miss discussions, I feel I will be missing a lot, sometimes if my network is available, then if it’s not available, then l would find a spot that would get network. [Male participant, 21 years] I think you have to change phones sometimes, because these phones they are not good at network they drop networks, they need to create a strong network coverage. [Male facilitator, 24 years] The challenges are that sometimes your phone’s battery is dead due to electricity cuts. Such a challenge may prevent you from participating in a discussion and sometimes network will be poor. [Female participant, 18 years] Mostly when I wanted to join the group, network would be an issue, so I would hotspot. If that doesn’t work again my facilitator used to come to my home: he had data, real data, and he would hotspot me. Then all the messages that were sent throughout the week, I would receive them, then I would respond to everything that happened during the week. [Female participant, 20 years] I think network is not a big problem for us, but the program we are using the Rocket chat maybe if we can convert it to WhatsApp, network would be easy for us, so the challenge is on the Rocket chat platform. [Female participant, 18 years] WhatsApp can draw attention of other people and the participant can be chatting to other people so Rocket chat is the best. [Male facilitator 24 years]	
	Participation from participants was variable	The difficulty moment is usually network problems, and also unavailability of other group members, some will be at work or school, it differs, sometimes there will be two or three members out of seven, it varies. [Female participant, 18 years] As for me, yes, I was participating, but sometimes I would get busy. But for guest sessions, I would not miss any. [Male participant, 20 years] Sometimes I write the messages on time but there are sometimes that I will be at school so I will only be able to log in later. [Male participant, 20 years] During the meeting, you would hear other people’s views, then agree with them or even disagree, unlike doing that after meeting time, if you say I agree or disagree with so and so’s view, it will be pointless because they will be outside the room, so when alone it would just be writing message. So, I think participating on the set time is the best because there will be exchange of ideas, and if you have someone that you agree with, you agree there and there. [Male participant, 21 years]	
**4. Implementation**			
	Participants enjoyed the Zvatinoda! groups	Being a Zvatinoda! participant is a very good thing because you share ideas with your age mates and share your experiences and hear about other people’s experiences and sharing ideas. [Female participant, 18 years] I just feel that happy that I’m one of those Zvatinoda! participants because I can share what I see in the community and what I experience. [Female participant, 23 years] So far everything has been perfect so I don’t wish for any changes, and I wish to be engaged more...everything has been superb. [Male facilitator, 24 years]	
	Participants valued group interactions	Okay it was a great experience. Why, because I was talking to young people like me, who have ideas like mine, and I think we could relate to each other. And we communicated well even when we were to use our own language you would not understand it, but we would understand each other. So, it was awesome. [Male facilitator, 23 years] Zvatinoda! is a group where you can feel comfortable because we can express our thoughts freely without any disturbance like in other groups, whereby if you say your point, even if its valid, someone will always crush it, but your point will be correct. Unlike in Zvatinoda! group we understand each other, even if your point is wrong, they don’t tell you in an offending way, but they do so in a way that you will feel comfortable. [Male participant, 20 years] I now have confidence to talk to others, to share about whatever will be happening and I am no longer shy to talk about my personal hygiene. [Male participant, 20 years] To appreciate diversified ideas, I have learnt to be patient I have learnt to create a free environment for everybody to air out their views without any fear. [Female facilitator, 23 years]	
	Participants valued having privacy	It is good to interact over the phone using pseudo names I feel safe...you will not be shy to send your view. [Male participant, 20 years] It’s a good thing because its protecting confidentiality, someone will just say what they want, the experiences they went through, without any fear of being fingered out. [Female facilitator, 23 years] In this generation we use nicknames more than we use our names so its normal to us. [Male participant, 20 years] I would have participated less [if I was using my real name]...Some of the experiences, I will be talking about personal experiences so I don’t want people to know what I have been through, what I have faced, because of privacy that I require I would narrate it as someone else’s information, and not let others know that those are my actual experiences. [Male participant, 21 years]	
	Participants valued using project phones	I felt like it was a responsibility like no other. It was scary having an organization phone, I felt having a huge responsibility that I had to take care of it to make sure it was safe but at the same time it was amazing because the project team managed to trust us and give us phones. [Female facilitator, 23 years] As for me, the phone was safe and secure because I only used it when participating, if not that it would be at home. [Female participant, 20 years]	
	Value in delivery led by young people and supported by adults	When it comes to health discussions, it needs to be peer to peer, we are the ones who face the situation, so we understand each other as peer to peer, rather than someone who is not in that situation. [Male participant, 20 years] We feel much free when contributing, we won’t be afraid of being judged because we see each other at the same level. [Female participant, 18 years] We look down upon her and forget that she is our leader, and she [the facilitator] can also forget that we are supposed to be having a discussion today. [Female participant, 18 years] It’s good to have someone who is of your own age because he understands you. But...we also need control from an older project person...Sometimes the facilitator may do wrong thing, so there is need for an Auntie who will correct him/her and tell him/her that what he/she is doing is wrong. And the peer facilitators I think have little knowledge on the topics, they need some mentorship from Auntie. [Female participant, 18 years] Like in our African culture, Aunties are known to understand the youth mostly so it’s easy to go and tell her why I have been through what I have been experiencing so I would say we need more Aunties in the group so that we feel free to talk to them. [Male participant, 20 years] Aunties are good, but some boys are shy to talk to Aunties, so we need both a male Uncle and a female Auntie [Male participant, 20 years] To have an Auntie is okay because we won’t misbehave, we will have discipline. [Female participant, 18 years]	
	The regular feedback to health care providers was not delivered as planned	For us young people to go and access those services, I think there should be a, mainly a something like a corner just for the youths, where only the youths can go, where there are young nurses, they understand us more than older people. Of course older people need to be there, but they should be few, health care workers of our age should be more because they understand us better, we understand each other and there should be campaigns to other youths, I know it will take time for what I have said to be implemented. But, for now, having a place for the youths only so for now the nurses they should speak to us nicely, they should understand us, not that you go to report your issue and they are like, “are you sure you have been raped”, or “You asked to be raped because of your dressing”. So, they have their own stress, no salary and they take out the frustration on us, no that won’t work. [Female participant, 18 years] Challenges, people are always judgmental, and it takes time for them to be non-judgmental. I come with my story and they look surprised and say, ‘Ah at your age?’ [Male participant, 21 years] The elderly nurses should stop judging, when they are dealing with a client, they should be professional, accept clients and not judge based on age. It won’t be easy for them to accept, they would say they are parents as well and they would treat us as their own children. What they forget is that early age is indulging in sexual relationship, they should teach them that protection is the way to go to avoid diseases and unwanted pregnancies. [Female participant, 23 years] Interviewer: Alright that’s fine, thank you, how do you think the health care workers will respond to your feedback? What will they do? Respondent: Where possible they will react and do what we want but I’m sure where not possible, after hearing what we want they will go and make slight changes that are favourable to us. [Female facilitator, 23 years] At least if we are talking to them as young people, telling them they might understand us because this information is coming from many young people, so we want to see the change. [Male facilitator, 24 years]	
	The peer facilitators developed through their role in the intervention	I have learnt leadership, commitment, communication, and I also learnt about sexual and reproductive health mostly, which was also the agenda of the project. [Male facilitator, 24 years] I have learnt not to be judgmental, and I have learnt to accept others, being someone who is there to guide people in our community from young people and be a bridge between the community and the rest of the youths, I think and health facility as well. [Male facilitator, 24 years]	
**5. Maintenance**			
	Participant engagement reduced toward the end of the intervention	All participants during the first weeks of Zvatinoda! discussion were very engaged and would be on Rocket Chat before I join to facilitate the group discussions but the last 2 to 3 weeks engagement reduced with few participants opening Rocket chat post meeting time or day to read through the discussion and share their views’ [Male facilitator, 23 years]	
	Participants and peer facilitators desired an in-person element	As for mobile platforms haaa, I think what would work is groups, live, like meeting the groups, live, physically, not through the phone, because of network. [Female participant, 18 years] Ah, I think we should meet in person then we talk in person then they [the guest speakers] answer all our questions because sometimes they could answer all our questions but sometimes, they could not because there would be so many messages, so I think the best is to meet physically then you raise your hand and say what you want then another person does the same. [Male participant, 20 years] I think the last topics were straining because it had been a very long time without physical meetings, without encouragement from the meetings. [Male facilitator, 23 years]	

^a^RE-AIM: Reach, Effectiveness, Adoption, Implementation, and Maintenance.

^b^SRH: sexual and reproductive health.

Participants attempted to manage the network challenges by using another phone, either their own or a peer facilitator’s, as a hotspot: “I sacrificed to buy my own data then I would hotspot the phone” [Female participant, 18 years]. Peer facilitators had to be flexible to accommodate varying network availability: “as facilitators, we have to use initiative sometimes, if the network poses to be a challenge we just adjust to a suitable time.” [Male peer facilitator, 23 years]. To address network issues, suggestions included changing the project phones or using WhatsApp as an alternative platform, which requires less robust network connectivity. Although WhatsApp does not provide anonymity and some participants felt that “WhatsApp is not safe” [Female participant, 18 years], it was repeatedly recommended as a solution:

WhatsApp is actually good because everyone loves WhatsApp, [and] anyone can afford data for WhatsApp and a smart phone in the urban settings

Male participant, 23 years

#### Participation Was Variable

This was the case largely due to inconsistent network connectivity and work or school commitments. As a result, some groups had very few active participants on certain weeks. On average, 5 participants actively engaged in discussions each week. Some participants and peer facilitators thereby thought the group size should be increased, thinking the groups were “too small because some of our participants are not participating” [Female participants, 18 years]. Peer facilitators tried different ways to encourage participation, including supporting each other to “try to create a free environment where everyone would be free to participate” [Female peer facilitator, 23 years].

### Implementation

#### Factors Affecting Intervention Acceptability

#### Participants Enjoyed the Zvatinoda! Groups

A significant majority (24/28, 86%) reported a very positive experience (Table 2), with one participant stating, “my experience in Zvatinoda! is excellent” [Male participant, 23 years]. A few participants, who had been involved in other youth groups, noted that “compared to other programs, I think this one is the best” [Male participant, 21 years]. The intervention overall met and exceeded participants’ expectations, with one participant expressing, “it’s what I expected, it’s even way, way more than I expected” [Male participant, 20 years].

#### Participants Valued Group Interactions

They gained significant insights through interactions with their peers, peer facilitators, and guest speakers.

I can say whatever I think of, and others can also share their views, which makes me also gain information

Female participant, 22 years

The “bond” developed among participants allowed them to feel comfortable and open, enabling them to “talk about anything that we want and what we think” [Female participant, 22 years]. Through these interactions, participants encouraged each other to adopt safer SRH practices. For instance, one participant shared, “I managed to encourage more guys to get circumcised citing that I have been circumcised to just go there and they went for circumcision.” [Male participant, 20 years].

#### Participants Valued Privacy

The use of pseudonyms in Rocket.Chat was highly appreciated, as it provided a sense of security and enabled participants to express themselves openly.

if you are not using pseudonym names you will be shy, but, then if you are using pseudonym names, I feel free to say anything I want

Male participant, 23 years

This privacy allowed participants to “feel safe” [Male participant, 23 years], and “participate to the fullest without fear of being pointed out by name that you said this and that” [Male participant, 20 years]. However, as participants grew more familiar and comfortable with each other, the need for such privacy diminished.

on the first days I was uncomfortable, but through continuous interaction I know that even if were to use our real names I was still going to feel the same way.

Female participant, 18 years

#### Participants Valued Using Project Phones

Participants valued and were motivated by receiving a project phone and data to participate in the chats. As one participant put it, “I was happy that I was trusted to be given a gadget” [Female participant, 23 years]. While most felt comfortable with the phone and reported “no security issues” [Male peer facilitator, 24 years], a few expressed concerns about “feared the phone breaking down or being stolen” [Male participant, 21 years]. When asked about the phone by family members, classmates, and friends, participants were pleased to explain:

I joined this program called Zvatinoda!. It is mainly focusing on the youth giving on sexual and reproductive health education

Male participant, 23 years

Although understanding, participants were saddened to return the phone at the end of the intervention: “it’s quite painful because I have been used to have a phone, it’s been a part of me, but it has to be done.” [Female peer facilitator, 23 years]

#### Value in Delivery Led by Young People and Supported by Adults

The facilitation of the chat groups by young people was considered successful. Participants felt that “a facilitator who is of the same age with us understands us, and he knows the situations that we meet” [Female participant, 18 years]. Having a peer facilitator of a similar age allowed participants to “feel very free to say what we want; we speak our real views” [Female participant, 22 years]. However, participants also recognized the challenge that “that other team members may not respect the facilitator because we are on the same age” [Female participant, 22 years]. The peer facilitators were occasionally unreliable, arriving late to the chats or altering the scheduled times. The presence of an older moderator, often described as an “Auntie figure,” provided the necessary gravitas to the chat groups.

As young people, as youths, sometimes we might disrespect [our peer facilitator] and not get into the Rocket.Chat. But the Auntie is there to guide us: if we fail to get into the Rocket.Chat on time she is there to push us. When we have conflicts and fail to agree she is there to harmonize us.

Female participant, 22 years

The Auntie figure played a critical role “in making us become committed,” providing correct information, and also “to listen to our grievances” [Male participant, 21 years].

#### Factors Influencing Implementation Fidelity: The Regular Feedback to Health Care Providers Was Not Delivered As Planned

COVID-19 restrictions on gatherings prevented the scheduled in-person feedback meetings between young people and health care providers. Instead of the planned monthly feedback sessions, all feedback was consolidated into a single in-person meeting held after the completion of the mobile phone–based chat groups. At this in-person meeting, the peer facilitators summarized the key messages from each discussion topic. They compiled 1-page summaries that included key points from the chats on various health topics, encompassing young people’s health knowledge, needs, commitments, and commonly used slang or “youth speak” related to each topic. Participants had numerous suggestions for improving health services for young people. They recommended that “health services should be non-judgemental” [Male participant, 21 years] and that “nurses should undergo training for treating us with care” [Male participant, 23 years]. However, they also recognized that implementing some of these changes could be challenging. Perceived resistance from health care providers was noted:

these health service providers they would say ‘we have our own way of doing things so you cannot come and tell us what to do on our job’

Male participant, 21 years

The peer facilitators also created a music video and a spoken word performance highlighting key messages about youth health service needs. These were shared both at the feedback meeting and online. The feedback meeting provided an opportunity for the peer facilitators to directly present to health care professionals the results of the mobile phone–based chats, conveying what young people want from health services:

It’s quite a challenge but at the same time it’s an opportunity, that feeling which says I have stood in front of people telling them this and that, people who have studied this, but this time we are saying even though you studied it this is what we want.

Female peer facilitator, 23 years

#### Youth Capacity Building Through Intervention Implementation: The Peer Facilitators Developed Through Their Role in the Intervention

The peer facilitators themselves developed skills and “learnt personally” [Female peer facilitator, 23 years] through the implementation of the intervention:

It’s a good thing to be a facilitator, as in you gain a lot of things, for instance we had leadership training workshops, being taught to be a facilitator and I gained so much from there.

Female peer facilitator, 23 years

The peer facilitators described valuing “interacting with people” and learning to “accept diversified ideas” [Female peer facilitator, 23 years] and “not be judgemental” [Male peer facilitator, 24 years]. Through the training and experience facilitation, the peer facilitators noted that their “communication skills improved” and that they “have learnt to be leader[s]” [Male peer facilitator, 23 years]. However, when the intervention was ending, the peer facilitators expressed concerns about short-term interventions for young people, desires for continued engagement, and a fear of abandonment by programs.

### Maintenance

#### Participant Engagement Reduced Toward the End of the Intervention

Participant engagement on Rocket.Chat declined over the 12 weeks of the intervention, as noted by peer facilitators and the “Auntie.” Initially, peer facilitators did not need to prompt participants to join discussions. However, toward the end of the intervention, there was a greater need to send reminders before group discussion sessions and expert live chats. Perceptions of the length of the intervention varied, with some participants saying “it’s just right” [Male participant, 21 years], but others saying “they are too long, I would think if we would do 8 weeks” [Male participant, 23 years]. The peer facilitators found it challenging to maintain engagement on the mobile platform over the course of the intervention, especially without an in-person component:

I think the last topics were straining because it had been a very long time without encouragement from physical meeting

Male peer facilitator, 24 years

#### Participants and Peer Facilitators Desired an In-Person Element

To maintain engagement, an in-person element was suggested by many, mixed with mobile phone–based elements: “we also want groups where we can meet live and have discussions physically not through the phone” [Female participant, 18 years]. This was felt to make the intervention more appealing and increase the trust between participants:

that is going to make us have a strong relationship then we actually participate more knowing our fellow participants I can trust them, so I have to say anything I want to say

Male participant, 23 years

Participants generally felt that after a few weeks of anonymity to build confidence and openness, they would be comfortable meeting in person:

sometimes we need physical meetings to discuss so that we get to know each other even though we are not using the pseudonym names.

Male participant, 23 years

## Discussion

The Zvatinoda! digital health intervention proved acceptable and feasible, demonstrating the potential for enhancing young people’s knowledge and health-seeking behaviors through peer-to-peer learning and anonymous expert discussions. Increased knowledge led to improved health-seeking behaviors and the sharing of information with peers and family members outside the intervention. Participants valued the group interactions, the privacy of the anonymous virtual environment, and the fact that the discussions were youth led and adult supported. The pilot study’s assessment of different modalities and the impact of feedback on young people’s health care needs to service providers was limited by COVID-19 restrictions, which prevented face-to-face meetings. This component of the intervention warrants further exploration.

This study supports the evidence that mobile phone–based interventions are feasible for addressing health care gaps among young people in Southern Africa [30]. The COVID-19 pandemic has significantly increased the demand for and development of digital interventions aimed at promoting adolescent SRH globally [31]. Given the high rate of phone ownership in Zimbabwe [16], digital interventions have the potential to reach a broad and diverse population of young people. However, as other studies have shown, network challenges and disparities in access among rural young people can be significant barriers to facilitating access and participation [30]. This study contributes to the literature by emphasizing the added value of the anonymity provided by the Rocket.Chat platform and the effectiveness of mobile phone–based interventions in including a diverse group of young people. The findings suggest the benefits of a hybrid approach, combining mobile phone and in-person interventions. Initially, online anonymity can help build trust, while subsequent in-person interactions can strengthen relationships and engagement.

This study contributes to the evidence base by highlighting not only the value of peer-to-peer learning for young people [30], but also the critical role of adult mentorship and access to experts in enhancing youth-led interventions [32]. Learning through the Zvatinoda! intervention was facilitated by peer-to-peer interactions, which were significantly supported by adult supervision and expertise. This support came through the adult guest expert sessions and the oversight of the chat discussions. While we advocate for youth-led interventions [33], our findings underscore the importance of adult mentorship and involvement in such initiatives. Adult support and mentorship not only enhance the effectiveness of interventions but also provide essential safeguards for younger leaders, who often face similar pressures and vulnerabilities as the target population.

While the mixed methods evaluation approach provided an in-depth understanding of the feasibility of the Zvatinoda! intervention, several limitations were noted. COVID-19 restrictions on gatherings constrained our ability to conduct the planned regular feedback sessions between young people and health care providers. Consequently, the intervention included only 1 feedback session between participants, peer facilitators, and health care workers, rather than the multiple sessions originally planned. A more regular feedback loop between young people and health care providers was considered both unusual and beneficial, warranting further investigation. Additionally, self-reported outcomes may have been influenced by social desirability bias. Lastly, as this was a feasibility study, the intervention reached only a small number of young people. Consequently, quantitative analysis was limited to descriptive statistics due to the small sample size. This also constrained our assessment of reach as a construct, so the results primarily address the potential reach of the intervention if scaled up.

The Zvatinoda! intervention demonstrates promise as a digital approach to engaging young people in Zimbabwe, enhancing their health knowledge and health-seeking behavior. The key elements contributing to its success are mobile phone–based delivery, youth-led and adult-supported peer-to-peer and expert learning, and improvements in both knowledge and health-seeking behaviors. Further development, implementation, and evaluation of the intervention are needed. Potential improvements include incorporating in-person discussions once virtual group members have established a bond and addressing network challenges through updates to phones, network settings, or app platforms.

## Abbreviations

RE-AIM: Reach, Effectiveness, Adoption, Implementation, and Maintenance
SRH: sexual and reproductive health

## References

[1] Patton GC, Sawyer SM, Santelli JS, Ross DA, Afifi R, Allen NB, Arora M, Azzopardi P, Baldwin W, Bonell C, Kakuma R, Kennedy E, Mahon J, McGovern T, Mokdad AH, Patel V, Petroni S, Reavley N, Taiwo K, Waldfogel J, Wickremarathne D, Barroso C, Bhutta Z, Fatusi AO, Mattoo A, Diers J, Fang J, Ferguson J, Ssewamala F, Viner RM (2016). Our future: a Lancet commission on adolescent health and wellbeing. The Lancet.

[2] World Health Organization (WHO) (2017). Health for the World's Adolescents: A Second Chance in the Second Decade.

[3] World Health Organization (WHO) (2017). Global Accelerated Action for the Health of Adolescents (AA-HA!). WHO.

[4] Zimbabwe National Statistical Agency (ZIMSTAT) (2022). Zimbabwe 2022 Population and Housing Census.

[5] Zimbabwe National Statistics Agency (ZNSA) (2015). Zimbabwe Demographic and Health Survey.

[6] Busza J, Dauya E, Bandason T, Mujuru H, Ferrand RA (2014). "I don't want financial support but verbal support." How do caregivers manage children's access to and retention in HIV care in urban Zimbabwe?. J Int AIDS Soc.

[7] Chingono R, Mackworth-Young CRS, Ross D, Tshuma M, Chiweshe T, Nyamayaro C, Sekanevana Cuthbert, Doyle Aoife M, Weiss Helen A, Kohl Kid, Mangombe Aveneni, Madzima Bernard, McHugh Grace, Ferrand Rashida A (2021). Designing routine health checkups for adolescents in Zimbabwe. J Adolesc Health.

[8] World Health Organization (WHO) (2002). Global consultation on adolescent friendly health services: a consensus statement. WHO.

[9] Mazur A, Brindis CD, Decker MJ (2018). Assessing youth-friendly sexual and reproductive health services: a systematic review. BMC Health Serv Res.

[10] James S, Pisa PT, Imrie J, Beery MP, Martin C, Skosana C, Delany-Moretlwe S (2018). Assessment of adolescent and youth friendly services in primary healthcare facilities in two provinces in South Africa. BMC Health Serv Res.

[11] Kim B, White K (2017). How can health professionals enhance interpersonal communication with adolescents and young adults to improve health care outcomes?: systematic literature review. International Journal of Adolescence and Youth.

[12] Ministry of Health and Child Care (MOHCC) (2016). The National Health Strategy for Zimbabwe 2016-2020. World Bank.

[13] Ippoliti NB, L'Engle K (2017). Meet us on the phone: mobile phone programs for adolescent sexual and reproductive health in low-to-middle income countries. Reprod Health.

[14] Feroz AS, Ali NA, Khoja A, Asad A, Saleem S (2021). Using mobile phones to improve young people sexual and reproductive health in low and middle-income countries: a systematic review to identify barriers, facilitators, and range of mHealth solutions. Reprod Health.

[15] Huang K, Kumar M, Cheng S, Urcuyo AE, Macharia P (2022). Applying technology to promote sexual and reproductive health and prevent gender based violence for adolescents in low and middle-income countries: digital health strategies synthesis from an umbrella review. BMC Health Serv Res.

[16] Doyle AM, Bandason T, Dauya E, McHugh G, Grundy C, Dringus S, Dziva Chikwari C, Ferrand RA (2021). Mobile phone access and implications for digital health interventions among adolescents and young adults in Zimbabwe: cross-sectional survey. JMIR Mhealth Uhealth.

[17] Laidlaw R, Dixon D, Morse T, Beattie TK, Kumwenda S, Mpemberera G (2017). Using participatory methods to design an mHealth intervention for a low income country, a case study in Chikwawa, Malawi. BMC Med Inform Decis Mak.

[18] Thabrew H, Fleming T, Hetrick S, Merry S (2018). Co-design of eHealth interventions with children and young people. Front Psychiatry.

[19] Glasgow RE, Vogt TM, Boles SM (1999). Evaluating the public health impact of health promotion interventions: the RE-AIM framework. Am J Public Health.

[20] Glasgow RE, Harden SM, Gaglio B, Rabin B, Smith ML, Porter GC, Ory MG, Estabrooks PA (2019). RE-AIM planning and evaluation framework: adapting to new science and practice with a 20-year review. Front Public Health.

[21] Rocket.Chat.

[22] Simpson N, Kydd A, Phiri M, Mbewe M, Sigande L, Gachie T, Ngobeni M, Monese T, Figerova Z, Schlesinger H, Bond V, Belemu S, Simwinga M, Schaap A, Biriotti M, Fidler S, Ayles H (2021). Insaka: mobile phone support groups for adolescent pregnant women living with HIV. BMC Pregnancy Childbirth.

[23] Atujuna M, Simpson N, Ngobeni M, Monese T, Giovenco D, Pike C, Figerova Z, Visser M, Biriotti M, Kydd A, Bekker L (2021). Khuluma: Using participatory, peer-led and digital methods to deliver psychosocial support to young people living with HIV in South Africa. Front Reprod Health.

[24] Abu-Lughod L (1986). Veiled Sentiments: Honor and Poetry in a Bedouin Society.

[25] Chimedza D, Webb K, Chitiyo V, Zidana S, Mukotekwa T, Kanhongo T (2015). Reaching young people with mobile technology for sexual and reproductive health interventions: experiences from voices and choices project in Manicaland Province, Zimbabwe.

[26] Charashika P, Mackworth-Young C, Mangombe A, Simpson N, Kydd A, Webb K (2021). Zvatinoda! (What we want!): participatory methods for community-led formation of a youth advisory research panel in urban Zimbabwe.

[27] Webster TR, Mantopoulos J, Jackson E, Cole-Lewis H, Kidane L, Kebede S, Abebe Y, Lawson R, Bradley EH (2011). A brief questionnaire for assessing patient healthcare experiences in low-income settings. Int J Qual Health Care.

[28] Nsanya MK, Atchison CJ, Bottomley C, Doyle AM, Kapiga SH (2019). Modern contraceptive use among sexually active women aged 15-19 years in North-Western Tanzania: results from the Adolescent 360 (A360) baseline survey. BMJ Open.

[29] Stangl A, Lilleston P, Mathema H, Pliakas T, Krishnaratne S, Sievwright K, Bell-Mandla N, Vermaak Redwaan, Mainga T, Steinhaus M, Donnell D, Schaap A, Bock P, Ayles H, Hayes R, Hoddinott G, Bond V, Hargreaves J, HPTN 071 (PopART) Study Team (2019). Development of parallel measures to assess HIV stigma and discrimination among people living with HIV, community members and health workers in the HPTN 071 (PopART) trial in Zambia and South Africa. J Int AIDS Soc.

[30] Bergam S, Sibaya T, Ndlela N, Kuzwayo M, Fomo M, Goldstein MH, Marconi VC, Haberer JE, Archary M, Zanoni BC (2022). "I am not shy anymore": A qualitative study of the role of an interactive mHealth intervention on sexual health knowledge, attitudes, and behaviors of South African adolescents with perinatal HIV. Reprod Health.

[31] Cornelius JB, Smoot JM (2022). The impact of technology on adolescent sexual and reproductive needs. Int J Environ Res Public Health.

[32] Stangl AL, Mwale M, Sebany M, Mackworth-Young CR, Chiiya C, Chonta M, Clay S, Sievwright K, Bond V (2021). Feasibility, acceptability and preliminary efficacy of Tikambisane ('let's talk to each other'): a pilot support group intervention for adolescent girls living with HIV in Zambia. J Int Assoc Provid AIDS Care.

[33] Oliveras C, Cluver L, Bernays S, Armstrong A (2018). Nothing about us without RIGHTS-meaningful engagement of children and youth: from research prioritization to clinical trials, implementation science, and policy. J Acquir Immune Defic Syndr.

